# Perceived Health Status among Older Adults with Intellectual and Developmental Disabilities

**DOI:** 10.1093/geroni/igaf122.3991

**Published:** 2025-12-31

**Authors:** Jihyeong Jeong, Changhyun Kim, Sehyun Baek, Amanda Lehning

**Affiliations:** University of Maryland Baltimore, Baltimore, Maryland, United States; Busan National University, Busan, Republic of Korea; New York University, New York, New York, United States; University of Maryland Baltimore, Baltimore, Maryland, United States

## Abstract

With growing interest in the age-friendly community (AFC) movement, empirical research has increasingly explored the associations between AFC characteristics and various health outcomes. However, prior studies have primarily focused on the general older population, with limited attention given to more vulnerable subgroups, particularly older adults with intellectual and developmental disabilities (IDD). Using the National Core Indicators® - Intellectual and Developmental Disabilities in-person survey 2022-2023 data, we analyzed the association between AFC indicators and self-rated health among older adults with IDD aged 55 or older who were living in the community (N = 688). The AFC features analyzed are: (a) housing, (b) outdoor spaces and buildings, (c) transportation, (d) social participation and inclusion, (e) health and wellness, and (f) volunteering and civic engagement. Perceived health status was measured using a single item: “Overall, how would you describe your health?” We performed bivariate and logistic regression analyses controlling for covariates. Our study demonstrated significant associations between better self-rated health and housing satisfaction (OR = 3.13, p = .012), access to adequate transportation (OR = 1.94, p = .019), and social participation (OR = 1.15, p = .001). Based on the findings, we suggest that housing satisfaction for older adults with IDD should ensure cognitively accessible residential environments. Transportation services should address additional challenges that they encounter (e.g., understanding complex routes, coping with unexpected changes, and communication barriers). Promoting social participation should entail cognitive accessibility, trained support staff, and creating inclusive community environments that understand and accommodate communication difficulties.

